# Shoulder impingement: various risk factors for supraspinatus tendon tear: A case group study

**DOI:** 10.1097/MD.0000000000028575

**Published:** 2022-01-21

**Authors:** Rani G. Ahmad

**Affiliations:** Radiology Department, King Abdulaziz University Hospital, Faculty of Medicine, King Abdulaziz University, Jeddah, Saudi Arabia.

**Keywords:** Jeddah, risk factors, shoulder impingement, supraspinatus tendon, tear

## Abstract

A total of 680 cases of monolateral shoulder pain and functional impairment were included, and Chi-Squared tests was incorporated to test for possible associations.

No relation between impingement syndrome and potential risk factors was found, such as presence of down slopping (*P* = .083), presence of ossification acromiale *(P* *=* .102), presence of calcific tendinitis *(P* *=* .144), types of acromion (I [*P* = .600], II [*P* = .536], III [*P* = .633] and IV [*P* = .832]) and grade of acromioclavicular degenerative changes (mild [*P* = .077], moderate [*P* = .111], and severe [*P* = .700]). However, a significant relationship was uncovered between impingement syndrome and risk factors such as gender (X^2^ = 7.004, *df* = 1, *P* = .08) (where females were more prone), history of shoulder dislocation (X^2^ = 19.440, *df* = 1, *P* = .001), presence of supraspinatus tendon tear or tendinopathy (X^2^ = 69.344, *df* = 1, *P* = .001) and supraspinatus complete tear (X^2^ = 13.593, *df* = 1, *P* = .001). A significant relationship was found between the type of supraspinatus pathology and factors such as gender (female more prone) (X^2^ = 34.719, *df* = 3, *P* = .01), presence of down slopping (X^2^ = 57.765, *df* = 3, *P* = .01), history of shoulder dislocation (X^2^ = 148.880, *df* = 3, *P* = .001), type III of the acromion (X^2^ = 12.979, *df* = 3, *P* = .005), presence of acromioclavicular generative changes mild (X^2^ = 76.408, *df* = 3, *P* = .001) and moderate (X^2^ = 29.697, *df* = 3, *P* = .001), and acromiohumeral distance of ≤3 mm (X^2^ = 18.915, *df* = 3, *P* = .001), 3.1 to 6 mm (X^2^ = 13.212, *df* = 3, *P* = .004), and 9.1–12 mm (X^2^ = 15.066, *df* = 3, *P* = .002). Overall, the Magnetic Resonance Imaging results yielded high sensitivity for detecting full-thickness supraspinatus tears.

Considering the findings, this study may help radiologists understand the salient risk factors and identify which factors are mainly responsible for supraspinatus tendon tears and the respective grade of tear (articular partial, bursal partial, complete, or tendinopathy).

## 1 Introduction

Rotator cuff disease is one of the most common causes of shoulder pain, although there is uncertainty regarding the various options for effective treatment.^[1]^ In recent times, the detection of even small tears has become essential since they have been shown to progressively lead to necessary surgery to mitigate shoulder pain.^[2]^ Traditional magnetic resonance imaging (MRI) and ultrasonography are relatively accurate for the identification of full-thickness tears and a similar sensitivity for detecting partial-thickness tears.^[3]^ Impingement is the primary cause of rotator cuff pathologies; the supraspinatus tendon is usually the affected tendon because of anatomical structure, the tendon that passes under the acromion.^[4]^

The humeral head, coracoacromial ligament, acromioclavicular joint, and the surface of the anterior third of the acromion define the subacromial space. The supraspinatus tendons, subacromial bursa, the capsule of the shoulder joint, and long head of the biceps brachii tendon are the tissues that occupy the subacromial space.^[5]^ Subacromial impingement syndrome (SAIS) influences any or all of these structures. It is an encroachment of the subacromial tissues due to the subacromial space's narrowing. The narrowing of space characterizing SAIS has been described by 2 predominant mechanistic theories.^[6]^ The first is extrinsic impingement, where degeneration and inflammation of the tendon occur because of mechanical compression by some external structure to the tendon. The second is intrinsic impingement, where complete- or partial-thickness tendon tears form due to a degenerative process through time with tension overload, the trauma of the tendons, or prolonged overuse.^[7]^ Acromial shapes, abnormal kinematics of the joint, downsloping presence, ossification acromiale, acromioclavicular degenerative changes, and inferior osteophytes are understood risks that can lead to impingement.^[3]^

MRI of the shoulder at 3.0T field strength is highly sensitive and accurate in detecting supraspinatus tendon tears and broadly exceeds the efficacy of arthroscopic analysis.^[8]^ Indeed, even MRI at 1.5T field strength is sensitive enough to detect full-thickness supraspinatus tendon tears, although not as effective in detecting some other tendon pathologies.^[8–11]^ A partial tear is detected when a tendon defect extends to either the bursal or articular surface or can be intra-tendinous.^[12]^ Initially, MRI sensitivity to detect partial-thickness tears ranged from 35% to 87%. However, with the technological advancement of MRI machines, sensitivity can now be as consistently high as 85%.^[12]^

## 2 Materials and methods

The study included shoulder MRI patients from January 1, 2017, and May 31, 2020, at King Abdulaziz University Hospital. The study was approved by the King Abdulaziz University, Saudi Arabia, under the Ref. No. 348–20. The study sample included all patients with suspected or diagnosed subacromial impingement syndrome, along with asymptomatic healthy patients. The presence of any risk factors was evaluated in all patients, and the status of the supraspinatus tendon was recorded. We excluded patients with surgical intervention or any adjacent pathology that may affect the subacromial space, such as tumors or fractures.

Two independent radiologists evaluated all analyses with a list of risk factors that evaluated its presence and effect on the supraspinatus tendon. The supraspinatus tendon was assessed with no specific guidelines, but the status of the tendon as found in the MRI images was clearly emphasized to be divided into tendinopathy, articular partial-thickness bursal partial thickness, complete tear, and normal anatomy. The supraspinatus tendon was scanned at parallel and perpendicular perspectives. The risk factors studied included the presence of downsloping, ossification acromiale, acromioclavicular degenerative changes, and inferior osteophytic formations.

MRI examinations were performed with the same protocols, using standard 3T MRI machines (Philips Achieva 3T MRI and Siemens MAGNETOM Trio 3.0T MRI machine manufactured in the Middle East). Examinations were performed on the shoulder using mild external rotation. This anatomic position optimally places the supraspinatus tendon perpendicular and parallel to the oblique sagittal imaging and oblique coronal planes.

**Figure 1:**
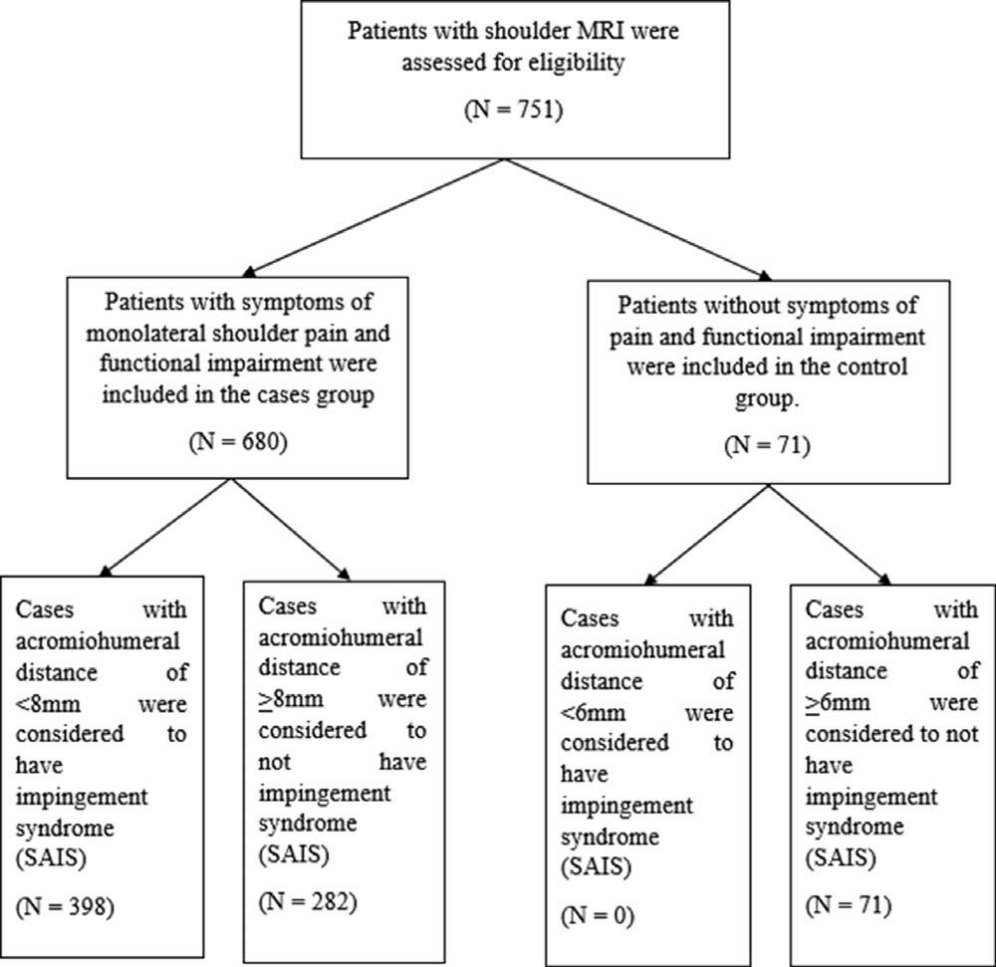
OA Guidelines Flow Diagram.

Data were reported as mean ± SD for continuous variables, and dichotomous and categorical variables were reported as percentages and frequencies. The Chi-Squared test was used for evaluating relationships between categorical data. A *P* value of <.05 was set as statistically significant (Fig. 1).

## 3 Results

Six eighty cases were included in the case group, and 71 cases were in the control group. Six hundred eighty patients with monolateral shoulder pain and functional impairment were included with a mean age of 48.66 ± 14.41 years, whereas the control group had a mean age of 36.25 ± 13.06 years. The gender ratio was equal (340 males and 340 females) in the case group. Out of 680 cases, 328 cases with type II acromion were detected, identifying this type as the most common of all types found (48.2%). The presence of downsloping was frequently reported in 30.58% of cases, while ossification acromiale was reported in 6.0% of the cases. Of the 680 patients in the case group, 17.79% reported moderate acromioclavicular degenerative changes (Table 1).

**Table 1:** Patient profile.

Variables	Cases group (n = 680)	Control group (n = 71)
Age (mean ± SD; range)	48.66 ± 14.41 (12–85)	35.89 ± 13.17 (13–66)
Gender		
Male	340 (50%)	40 (56.3%)
Female	340 (50%)	31 (43.7%)
Type of acromion		
I	295 (43.4%)	38 (53.52%)
II	328 (48.2%)	28 (39.43%)
III	27 (4.0%)	1 (1.40%)
IV	30 (4.4%)	4 (5.6%)
Presence of down sloping		
Yes	208 (30.58%)	0 (0%)
No	472 (69.41%)	71 (100%)
Presence of OS acromiale		
Yes	41 (6.0%)	1 (1.40%)
No	639 (94%)	70 (98.69%)
Presence of AC degenerative changes		
Mild	307 (45.14%)	13 (18.3%)
Moderate	121 (17.79%)	3 (4.2%)
Severe	22 (3.23%)	0 (0%)
None	230 (33.83%)	55 (77.5%)
Inferior osteophytes length (mean ± SD; range)	2.67 ± 1.27 (0.5–7)	–
Acromiohumeral distance	7.62 ± 1.52 (3–13)	8.95 ± 1.056 (6.9–12)
Supraspinatus tendon		
Normal	119 (17.5%)	70 (98.59%)
Partial tear	210 (30.88%)	0 (0%)
Complete tear	143 (21.02%)	0 (0%)
Tendinopathy	208 (30.58%)	1 (1.40%)
Partial supraspinatus tear (n = 210)		
Articular	147 (21.6%)	0 (0%)
Bursal	47 (6.9%)	0 (0%)
Both	117 (17.2%)	0 (0%)
Sub acromion subdeltoid Bursitis		
Large amount of fluid	36 (5.3%)	0 (0%)
Mild fluid	460 (67.6%)	6 (8.5%)
None	178 (26.2%)	65 (91.42%)
Presence of calcific tendinitis		
Mild	31 (4.6%)	0 (0%)
Severe	9 (1.3%)	0 (0%)
None	640 (94.1%)	71 (100%)
Presence of sign of chronic shoulder dislocation (n = 680)		
Yes	107 (15.7%)	0 (0%)
No	573 (84.3%)	71 (100%)

Patients with acromiohumeral distance of <8 mm were considered to have impingement syndrome when associated with significant clinical findings to warrant this classification. The relation between impingement syndrome and the risk factors were calculated using the Chi-Squared test (Table 2), which showed no link between factors such as presence of down slopping (*P* = .083), presence of ossification acromiale (*P* = .102), presence of calcific tendinitis (*P* = .144), type of acromion (type I [*P* = .600], type II [*P* = .563], type III [*P* = .633], type IV [*P* = .832]), and grade of acromioclavicular degenerative change mild [*P* = .077], moderate [*P* = .111], severe [*P* = .700]). Although statistically insignificant, type I acromion was present in 176 of 295 patients (59.7%), type II in 188 of 328 patients (57.3%), type III in 17 of 27 patients (63%), and type IV in 17 of 30 (56.7%) patients with impingement syndrome. However, a statistically significant relationship was found between factors such as the history of shoulder dislocation (X^2^ = 19.440, *df* = 1, *P* = .001), presence of supraspinatus tendon tear, or pathology (X^2^ = 69.344, *df* = 1, *P* = .001), and gender (X^2^ = 7.004, *df* = 1, *P* = .08). Indeed, 216 females (63.5%) had impingement syndrome compared to 182 males (53.5%). Compared with supraspinatus pathologies independently, impingement syndrome was not present significantly in 90 of 119 patients (75.6%) with no pathology (X^2^ = 73.576, *df* = 1, *P* = .001). By contrast, impingement was significantly present in 103 of 143 patients (72.0%) with complete supraspinatus tear (X^2^ = 13.593, *df* = 1, *P* = .001). However, partial tear (*P* = .089) and tendinopathy (*P* = .057) were not significantly associated with impingement syndrome.

**Table 2:** Relationship of impingement syndrome and various risk factors.

		Impingement Syndrome		Chi square		
Variables	N	Yes (N = 398)	No (N = 282)	Value (X^2^)	df	*P* value
Gender	680			7.004	1	.008
Male	340	182 (53.5%)	158 (46.5%)			
Female	340	216 (63.5%)	124 (36.5%)			
Presence of down sloping	680			3.003	1	.083
Yes	208	132 (63.5%)	76 (36.5%)			
No	472	266 (56.4%)	206 (43.6%)			
Presence of OS acromiale	680			2.670	1	.102
Yes	41	19 (46.3%)	22 (53.6%)			
No	639	379 (59.3%)	260 (40.7%)			
Presence of calcific tendinitis	680			2.130	1	.144
Yes	40	19 (47.5%)	21 (52.5%)			
No	640	379 (59.2%)	261 (40.8%)			
Presence of sign of chronic shoulder dislocation	680			19.440	1	.001
Yes	107	42 (39.3%)	65 (60.7%)			
No	573	356 (62.1%)	217 (37.9%)			
Types of acromion type	680					
I	295	176 (59.7%)	119 (40.3%)	.275	1	.600
II	328	188 (57.3%)	140 (42.7%)	.384	1	.536
III	27	17 (63%)	10 (37%)	.228	1	.633
IV	30	17 (56.7%)	13 (43.3%)	.045	1	.832
Presence of AC degenerative changes	680					
None	230	132 (57.4%)	98 (42.6%)	.185	1	.667
Mild	307	191 (62.2%)	116 (37.8%)	3.132	1	.077
Moderate	121	58 (52.1%)	58 (47.9%)	2.533	1	.111
Severe	22	12 (54.5%)	10 (45.5%)	.149	1	.700
Presence of Supraspinatus tendon tear or tendinopathy	680			69.344	1	.001
Yes	561	369 (65.8%)	192 (34.2%)			
No	119	29 (24.4%)	90 (75.6%)			
Type of Supraspinatus tendon tear	680					
Normal	119	29 (24.4%)	90 (75.6%)	73.576	1	.001
Partial tear	210	133 (63.3%)	77 (36.7%)	2.889	1	.089
Complete tear	143	103 (72.0%)	40 (28.0%)	13.593	1	.001
Tendinopathy	208	133 (63.9%)	75 (36.1%)	3.617	1	.057

The relation between type of supraspinatus tear and multiple risk factors was likewise analyzed using a Chi-Squared test, which showed no relation with factors such as the presence of ossification acromiale (*P* = .240) and presence of calcific tendinitis (*P* = .416) (Table 3). However, significant relationships were found between factors such as gender (X^2^ = 34.719, *df* = 3, *P* = .01)—where females were more prone to supraspinatus tear, presence of down slopping (X^2^ = 57.765, *df* = 3, *P* = .01), history of shoulder dislocation (X^2^ = 148.880, *df* = 3, *P* = .001), where 61 of 107 patients (57%) with a history of shoulder dislocation had no pathology and type III of the acromion (X^2^ = 12.979, *df* = 3, *P* = .005), and were 16 of 27 patients (59.3%) had a partial tear. A similar significant association between supraspinatus pathology was found with the presence of mild (X^2^ = 76.408, *df* = 3, *P* = .001) and moderate (X^2^ = 29.697, *df* = 3, *P* = .001) acromioclavicular generative changes, where 126 of 307 mild cases had a partial tear, and 47 of 121 moderate cases (38.8%) had a complete tear. An acromiohumeral distance of ≤3 mm (X^2^ = 18.915, *df* = 3, *P* = .001), 3.1 to 6 mm (X^2^ = 13.212, *df* = 3, *P* = .004), and 9.1 to 12 mm (X^2^ = 15.066, *df* = 3, *P* = .002) were significantly associated with pathologies. Acromiohumeral distance of ≦3 mm had 5 out of 5 cases (100%) with a complete tear. A distance of 6.1 to 9 mm had 33 out of 106 cases (33%) with a partial tear, 33 of 106 cases (33%) with a complete tear, a distance of 9.1 to 12 mm had 2 of 3 cases (66.7%) of tendinopathy present.

**Table 3:** Relationship between supraspinatus tear and tendinopathy and various risk factors.

		Type of supraspinatus tear				Chi-Squared test		
Variables	N	Normal/no tear (N = 119)	Partial tear (210)	Complete tear (N = 143)	Tendinopathy (208)	Value (X^2^)	*df*	*P* value
Gender	680					34.719	3	.001
Male	340	86 (25.3%)	101 (29.7%)	52 (15.3%)	101 (29.7%)			
Female	340	33 (9.7%)	109 (32.1%)	91 (26.8%)	107 (31.5%)			
Presence of down sloping	680					57.765	3	.001
Yes	208	8 (3.8%)	83 (39.9%)	65 (31.3%)	52 (25.0%)			
No	472	111 (23.5%)	127 (26.9%)	78 (16.5%)	156 (33.1%)			
Presence of OS acromiale	680					4.209	3	.240
Yes	41	3 (7.3%)	17 (41.5%)	9 (22.0%)	12 (29.3%)			
No	639	116 (18.2%)	193 (30.2%)	134 (21.0%)	196 (30.7%)			
Presence of calcific tendinitis	680					2.847	3	.416
Yes	40	10 (25.0%)	10 (25.0%)	6 (15.0%)	14 (35.0%)			
No	640	109 (17.0%)	200 (31.3%)	137 (21.4%)	194 (30.3%)			
Presence of sign of chronic shoulder dislocation	680					148.880	3	.001
Yes	107	61 (57.0%)	11 (10.3%)	4 (3.7%)	31 (29.0%)			
No	573	58 (10.1%)	199 (34.7%)	139 (24.3%)	177 (30.9%)			
Types of acromion type	680							
I	295	54 (18.3%)	85 (28.8%)	61 (20.7%)	95 (32.2%)	1.390	3	.708
II	328	56 (17.1%)	103 (31.4%)	71 (21.6%)	98 (29.9%)	.341	3	.952
III	27	0 (0.0%)	16 (59.3%)	5 (18.5%)	6 (22.2%)	12.979	3	.005
IV	30	9 (30.0%)	6 (20.0%)	6 (20.0%)	9 (30.0%)	4.025	3	.259
Presence of AC degenerative changes	680							
None	230	81 (35.2%)	49 (21.3%)	8 (3.5%)	92 (40%)	133.643	3	.001
Mild	307	15 (4.9%)	126 (41.0%)	79 (25.7%)	87 (28.3%)	76.408	3	.001
Moderate	121	21 (17.4%)	27 (22.3%)	47 (38.8%)	26 (21.5%)	29.697	3	.001
Severe	22	2 (9.1%)	8 (36.4%)	9 (40.9%)	3 (13.6%)	7.548	3	.056
Acromiohumeral distance	680							
≤3 mm	5	0 (0.0%)	0 (0.0%)	5 (100%)	0 (0.0%)	18.915	3	0.001
3.1–6 mm	106	8 (7.5%)	33 (31.3%)	33 (31.3%)	32 (30.2%)	13.212	3	0.004
6.1–9 mm	487	84 (17.2%)	155 (31.8%)	92 (18.9%)	156 (32.0%)	5.460	3	0.141
9.1–12 mm	79	26 (32.9%)	22 (27.8%)	13 (16.5%)	18 (22.8%)	15.066	3	0.002
>12 mm	3	1 (33.3%)	0 (0.0%)	0 (0.0%)	2 (66.7%)	3.278	3	0.351

One hundred eighty three cases reported inferior osteophytes size, classified into 5 groups according to size ranging from ≦1 mm to >4 mm. Table 4 shows the relationship between the size of the inferior osteophyte and factors such as impingement syndrome and type of supraspinatus pathology (tear and tendinopathy). It was found that inferior osteophyte size was not significantly associated with the presence of impingement syndrome (*P* = .367). None of the sizes of osteophyte ≤1 mm (*P* = .616), 1.1 to 2 mm (*P* = .075), 2.1 to 3 mm (*P* = .794), 3.1 to 4 mm (*P* = .191), and >4 mm (*P* = .103) was associated with supraspinatus pathology.

**Table 4:** Relationship between size of inferior osteophyte and other outcomes.

		Length of inferior osteophyte				
Variables	N	≤1 mm (N = 4)	1.1–2 mm (N = 86)	2.1–3 mm (N = 49)	3.1–4 mm (N = 26)	>4 mm (N = 18)
Impingement Syndrome	183					
Yes	114	3 (2.6%)	51 (44.7%)	31 (27.2%)	20 (17.5%)	9 (7.9%)
No	69	1 (1.4%)	36 (52.2%)	18 (26.1%)	6 (8.7%)	9 (13.0%)
*P* value^∗^ (X^2^, *df*)	.367 (4.150, 4)					
Type of Supraspinatus pathology	183					
Normal	15	2 (13.3%)	10 (13.3%)	4 (8.2%)	0 (0.0%)	1 (5.6%)
Partial tear	63	7 (46.7%)	28 (37.3%)	18 (36.7%)	8 (30.8%)	2 (11.1%)
Complete tear	65	4 (26.7%)	19 (25.3%)	19 (38.8%)	13 (50.0%)	10 (55.6%)
Tendinopathy	38	2 (13.3%)	18 (24.0%)	8 (16.3%)	5 (19.2%)	5 (27.8%)
*P* value^∗^ (X^2^, *df*)		.616 (1.794, 3)	.075 (6.901, 3)	.794 (1.030, 3)	.191 (4.757, 3)	.103 (6.181, 3)

## 4 Discussion

The occurrence of tears of the supraspinatus tendon is observed frequently in comparison with that of other rotator cuff tendons.^[13,14]^ Owing to the hypovascularity near the insertion of the supraspinatus tendon, specifically on its articular side, the partial-thickness tear is much higher on the supraspinatus tendon's undersurface.^[15]^ The objective of this study was to evaluate the various risk factors for the supraspinatus tendon in cases of SAIS.

Both genders were equally represented in this study. However, the role of gender in developing impingement was statistically significant, with females being more prone to impingement syndrome, partial or complete tendon tear, and tendinopathy. This finding contrasts with the results of several previous studies, which showed that male patients had a comparatively more significant number of shoulder lesions.^[16–18]^ However, Razmjou, Lincoln, Macritchie, et al^[19]^ posit that females are more severely affected by shoulder injuries or pathologies despite the higher prevalence in males.

An anterior or lateral down-sloping and a low-lying acromion are essential in developing subacromial impingement.^[20]^ However, in current research, the prevalence of downsloping in the presence of impingement syndrome was found statistically insignificant. Still, more specifically, statistical significance was reached with the presence of supraspinatus tendon tear or tendinopathy. This means the presence of supraspinatus tendon pathology may require the radiologist to look for the existence of down-sloping carefully. By contrast, the presence of down-sloping does not mean a significant association with the presence of the SAIS.

Ossification acromiale is effectively evaluated in upper axial images, where a low signal space is identified between the non-fused ossicle and the high-signal marrow of the distal acromion.^[21]^ The double acromioclavicular joint can detect it on the coronal oblique. The ossification acromiale is prevalent in between 1% and 15% of the general population.^[22]^ Previous studies have revealed the considerable risk in developing impingement when this relatively common variant is present.^[23,24]^ However, the current study's findings show no significant relationship with impingement syndrome, supraspinatus tear, or the presence of impingement symptoms. The failure to detect the considerable correlation of this relatively standard variant could be a product of the small sample size, and more studies are recommended to confirm or disconfirm a correlation.

The relationship between the type of acromion and supraspinatus tear was also significant where all the participants with type III had a partial tear (59.3%), complete tear (18.5%), or tendinopathy (22.2%). This means type III acromion has a higher chance of a concomitant supraspinatus pathology. This is in accordance with one study showing that type III is most significantly associated with full-thickness or a complete tear.^[25]^ However, another study (Kim et al) did not correlate acromion type and rotator cuff tears. The difference in findings between the studies could be because Kim et al^[26]^ carried out minimal participants.

The presence of acromioclavicular generative severity was also significant, where, with mild and moderate degenerative changes, there was a greater chance of having a partial or complete tear. It has been postulated that successful resection of acromioclavicular degeneration is necessary to treat rotator cuff tear.^[27]^ Thus, in the presence of supraspinatus tear, acromioclavicular degeneration should also be monitored to support recovery.

Calcific tendinitis was not correlated with impingement syndrome and type of supraspinatus pathology. However, calcific tendinitis and partial supraspinatus tears should be closely monitored, as the symptoms of one may mask the other, preventing detection.^[28]^

Chronic shoulder dislocation had a significant relationship with impingement syndrome: participants with a history of shoulder dislocation had less (39.2%) chance of getting impingement than participants with no history (62.1%). Likewise, participants with a history of shoulder dislocation had a greater chance of not having supraspinatus pathology than participants with no history that observed correlation with supraspinatus tears and tendinopathy. However, further study is required to more fully establish associations because previous studies have not generally considered shoulder dislocation when assessing risk factors for impingement.^[29–30]^ One study has shown that when shoulder instability is concomitant with impingement syndrome, it can cause significantly increased internal rotation.^[31]^ However, the study was limited to radiographical findings, excluding clinical findings.

The cases with reported inferior osteophytes were small in number. However, where present, the size of the inferior osteophyte did not correlate with the impingement syndrome or the type of supraspinatus pathology. This appears to contrast with 1 study, which suggests that inferior osteophytes are among the primary causes of impingement.^[4]^ The failure to detect the significant correlation is possibly owing to the small sample size of this relatively normal variant, and future studies are recommended to confirm or disconfirm an association.

Acromiohumeral distance correlated with supraspinatus pathology. The presence of ≤3 mm distance was 100% correlated with a complete tear. Cases with 6.1 to 9 mm distance had an equally high chance of partial and complete tear, and 9.1 to 12 mm distance had the highest association with tendinopathy. This acromiohumeral distance correlation is well known.^[32]^ However, patients with decreased distance should be closely monitored postoperatively as they pose a higher chance of rotator cuff re-tear.^[33]^

Further studies should be done to find the association of variants as a secondary cause of impingement that is not correlated with impingement syndrome and is correlated to supraspinatus pathologies—owing to the finding that supraspinatus is not correlated pathology is highly correlated with impingement.

## 5 Limitations

This study included all age groups; therefore, an actual risk factor related to age was not found. No randomization between the groups was done. Further, the failure to detect the significant correlation cold is because of the small sample size of variants such as OS acromiale, type 3, and 4 of the acromion. Also, the length of the inferior osteophyte in very few patients was measured.

## 6 Conclusion

This study found that females were more susceptible to shoulder impingement syndrome, supraspinatus tendinopathy, and supraspinatus tendon tear. The presence of supraspinatus tendon tear or tendinopathy was a high-risk factor for impingement. The prevalence of down-sloping in the presence of impingement syndrome was found statistically insignificant. Still, more particularly, a statistical significance was found in supraspinatus tendon tear or tendinopathy. The participants with no history of shoulder dislocation had a higher risk of impingement and tendinopathy—however, the less the acromiohumeral distance, the more significant the correlation with complete supraspinatus tear. The present study showed no correlation between ossification acromiale and impingement. Mild acromioclavicular degenerative changes were a risk factor for partial supraspinatus tendon tear, and moderate degenerative changes were a risk factor for complete tear. Calcific tendinitis and inferior osteophytes did not correlate with impingement or tendon pathology. Type of acromion was an associated risk factor, with type III being more susceptible to a partial tear. MRI scans showed a high sensitivity for detecting full-thickness supraspinatus tears.

## Author contributions

**Conceptualization:** Rani G. Ahmad.

**Methodology:** Rani G. Ahmad.

**Project administration:** Rani G. Ahmad.

**Supervision:** Rani G. Ahmad.

**Writing – original draft:** Rani G. Ahmad.

**Writing – review & editing:** Rani G. Ahmad.
